# A Case of Early-Stage Lower Uterine Segment Carcinoma With Hysteroscopic Observation of Visual Changes Induced by a Gonadotropin-Releasing Hormone (GnRH) Antagonist

**DOI:** 10.7759/cureus.74290

**Published:** 2024-11-23

**Authors:** Yoko Suzuki, Hidetaka Sato, Sakura Kataoka, Misato Ueda, Natsuki Nagashima, Asuka Yoshiara, Naoko Nakazawa

**Affiliations:** 1 Department of Obstetrics and Gynecology, Tokyo Metropolitan Police Hospital, Tokyo, JPN

**Keywords:** endometrioid cancer, gnrh antagonist, hysteroscopy, lower uterine segment cancer, lus

## Abstract

Endometrial carcinomas in the isthmus are called lower uterine segment (LUS) cancers. It is a rare location among uterine cancers and is known to be associated with Lynch syndrome, which tends to occur at a young age. Preoperative diagnosis may be difficult due to its anatomical location, and the prognosis is poorer than that of uterine cancer in general. Gonadotropin-releasing hormone (GnRH) antagonists, through their strong suppression of sex hormone release, prove advantageous as preoperative medication, ensuring optimal visualization in hysteroscopic surgery. We have encountered a case of early-stage LUS cancer diagnosed postoperatively after hysteroscopic surgery modified by GnRH antagonist treatment. In this report, we describe the characteristics of the visual findings of hysteroscopy, along with a literature review. The patient, a 32-year-old nulliparous woman, was referred to our facility after multiple endometrial polyps with atypical blood vessels were detected during a hysteroscopy performed during infertility treatment at her primary clinic. As endometrial cytology was negative, hysteroscopic tumor resection was performed after a short course of GnRH antagonist therapy. Lesions observed during hysteroscopy were broad-based with a scar-like white appearance and no abnormal blood vessels. Malignancy was not suspected until postoperative pathology confirmed grade 2 endometrioid cancer. For early detection of LUS cancer, it is worth familiarizing with its findings under hysteroscopy and the changes in appearance related to hormonal changes.

## Introduction

Endometrial carcinomas in the isthmus are referred to as lower uterine segment (LUS) cancers, accounting for about 3% of all uterine cancers [1]. Endometrial cytology is a simple and minimally invasive test for screening endometrial malignancies in outpatient settings. However, there is a possibility of inadequate sampling, which may lead to missing lesions. On the other hand, gonadotropin-releasing hormone (GnRH) antagonists, which inhibit pituitary GnRH receptors and strongly suppress the release of downstream sex hormones, are commonly used as conservative therapy before surgery for gynecological conditions influenced by estrogen, such as endometriosis, uterine fibroids, or even endometrial polyps. 

Hysteroscopy is widely used to treat uterine polyps, traditionally focusing on benign lesions. Recently, its application has expanded to include the diagnosis of endometrial malignancy. We encountered a case where endometrial cytology was negative; however, after a short course of GnRH antagonist therapy followed by hysteroscopic mass resection, the patient was postoperatively diagnosed with grade 2 endometrioid carcinoma. Unexpectedly, the LUS’s visual changes induced by GnRH antagonist therapy were observed. The atypical vasculature initially noted in the previous clinic had disappeared, leaving only a circumferential whitish lesion, making intraoperative malignancy diagnosis challenging. However, upon reviewing the surgical video with a focus on potential malignancy, several features that differed from typical hormone-induced endometrial atrophy or scarring were identified.

## Case presentation

The patient was 32, G0P0, 160cm, 50kg. She has a medical history of bipolar disorder controlled with medication and asthma, diagnosed one year ago. Her menarche occurred at age 10, and her menstrual cycles have been regular, with a 28-day cycle lasting seven days without abnormal bleeding. Menstrual flow is normal, and she experiences mild dysmenorrhea. Additional information includes a general allergy to animals, as well as a history of occasional alcohol consumption and smoking. Family history is significant for diabetes in her grandfather and cardiovascular disease in her grandmother. 

The patient was keen on conceiving and had been undergoing intrauterine insemination (IUI) at a previous clinic. Three months into treatment, a hysteroscopy was performed due to suspected endometrial polyps, during which atypical vasculature associated with an endometrial polyp was noted. One month later, she was referred to our hospital. At her initial visit, we performed endometrial cytology, which yielded a negative result. Given this outcome, the decision was made to use the GnRH antagonist relugolix (Relumina) for 19 days until the scheduled surgery. Five months after the initial suspicion of polyps, hysteroscopic resection of the endometrial polyp (TCR-p) was planned at our hospital.

**Figure 1:**
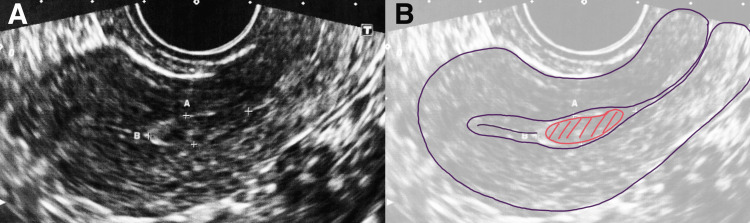
Preoperative transvaginal ultrasound A: Preoperative transvaginal ultrasound revealed that the polyp had already become indistinct and significantly reduced in size. B: Schermer depicts the endometrial polyps located on the lower segment of the uterine body.

On the day of the surgery, the preoperative transvaginal ultrasound revealed that the polyp had already become indistinct and significantly reduced in size (Figure 1).

**Figure 2:**
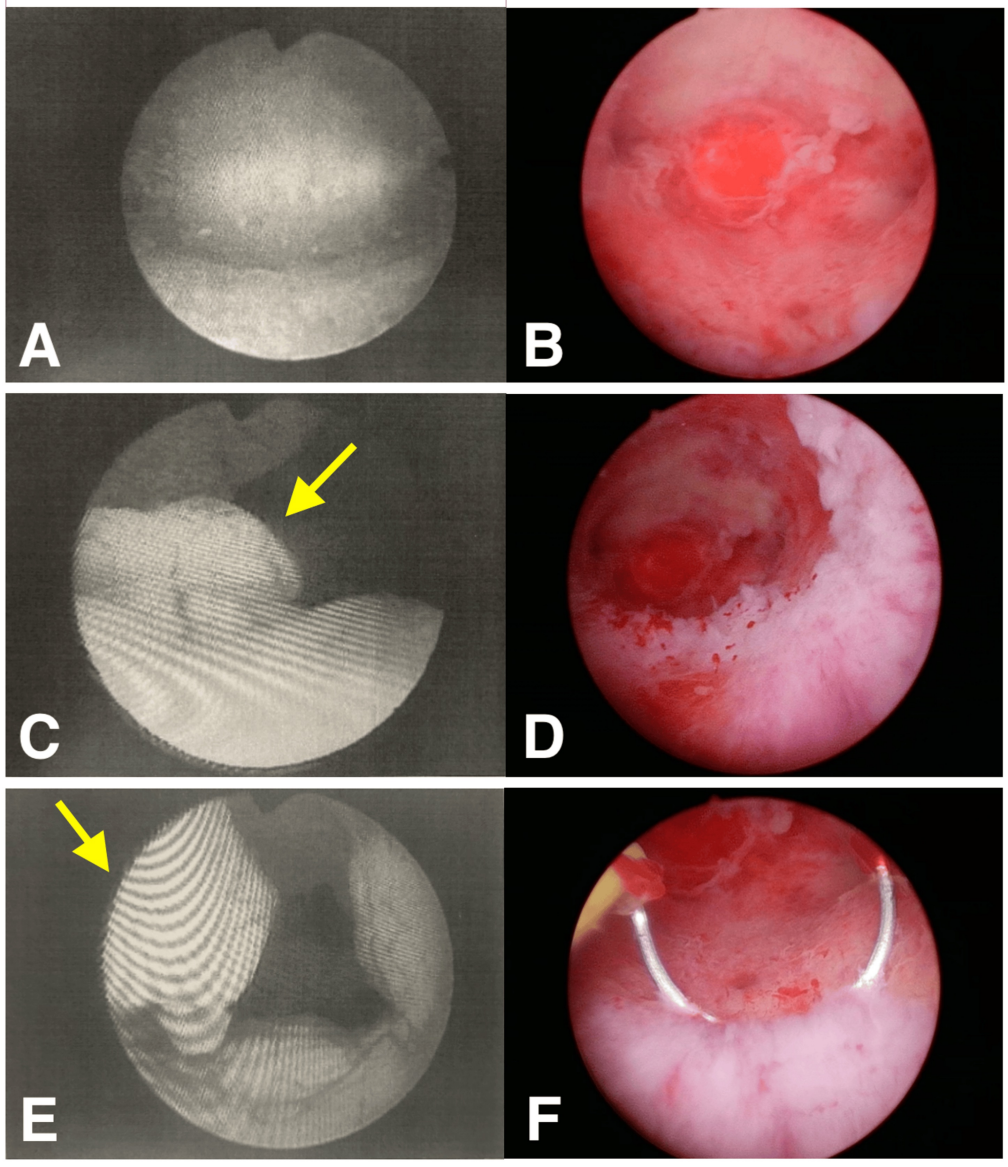
Hysteroscopic findings before and after administration of the GnRH antagonist. The left column shows the hysteroscopic findings from a previous physician. Endometrial polyps with atypical vessels could be seen. The right column shows the observation during our hysteroscopy after GnRH antagonist administration. The lesions were broad-based, scar-like white, without abnormal blood vessels. A and B: Fundus of the uterus, no specific occupying lesions, floating desquamation is more evident under hysteroscopy during our procedure. C and D: Comparison in the visual field of polypoid tissue in the lower segment of the uterus, where atypical vessels were obscured and protruding polyps (yellow arrow) were absent after modification with the GnRH antagonist. E and F: The polypoid tissue has disappeared (yellow arrow), leaving white scarred tissue, and is excised through a trans-hysteroscopic energy device. GnRH: Gonadotropin-releasing hormone

Intraoperative findings showed a normal uterine fundus and bilateral tubal ostia, while the posterior LUS presented with flat, white, scar-like changes. No atypical vasculature was observed, and the pronounced endometrial polyp noted in the previous clinic's images was not visible (Figure 2).

**Figure 3:**
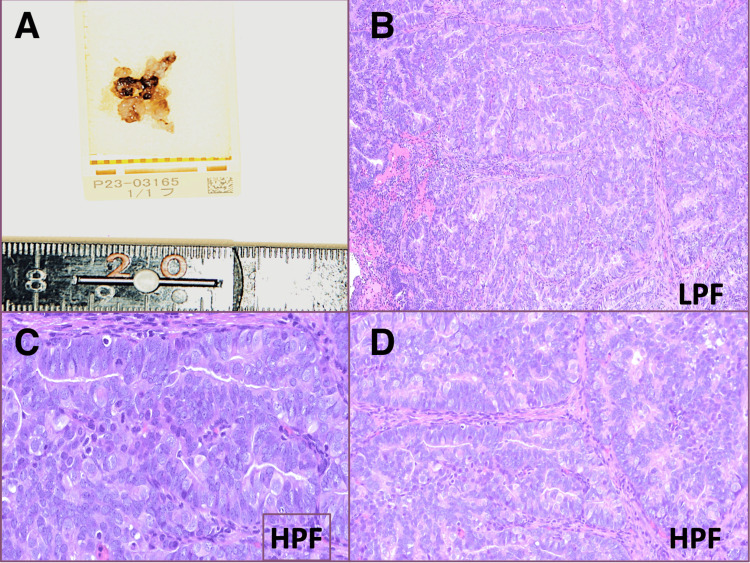
Pathologic findings A: Gross pathology specimens of resected tissue. B: Histology of low-power field presents moderately differentiated glands with less than half of the solid growth, consistent with endometrioid carcinoma grade 2 diagnosis (H&E, x100). C and D: Moderate nuclear atypia and visible mitotic activity under the microscope in the high-power field (H&E, x400).

The scar-like white area on the posterior wall was resected without residuals using monopolar cautery in the usual manner, ensuring the smooth lining of the endometrium before concluding the procedure. A bipolar cautery device was not accessible at the time of surgery. The surgery lasted 15 minutes and caused minimal blood loss. The specimen histologically presents as moderately differentiated glands with less than half of the solid growth, moderate nuclear atypia, and visible mitotic activity, consistent with the endometrioid carcinoma grade 2 diagnosis (Figure 3).

The patient, with a strong desire to preserve fertility, was promptly referred to a university hospital. Pathological analysis of the total curettage performed there indicated atypical hyperplasia. However, a reevaluation of the specimen obtained during our surgery confirmed a diagnosis of grade 2 endometrial carcinoma. As a result, conservative therapy such as medroxyprogesterone acetate therapy was deemed inappropriate and warrants further shared decision-making in treatment strategies, including genetic counseling.

## Discussion

Typically, we perform pathological diagnosis of the endometrium using a brush-type catheter for cytological diagnosis and a suction-type catheter or curette for histological diagnosis. In cases where malignancy is not strongly suspected, we opt for cytology with the brush-type catheter, which causes less discomfort during the procedure. A report revealed that the diagnostic sensitivity of two modalities, cytology vs. biopsy, was 24/33 (72.7%) vs. 28/33 (84.8%) for grade 2/3 endometrioid carcinomas and 44/77 (57.1%) vs. 56/77 (72.7%) when considering all grades (G1 and G2/3) [2]. Thus, the risk of false negatives and variability in sensitivity due to procedural and diagnostic errors is considerable. This is particularly evident in abnormalities in the LUS, where inadequate contact between the brush or pipette and the affected area can result in it being overlooked [3].

Preoperative use of dienogest, progestin, GnRH agonists, or antagonists can keep the endometrium in the thin phase, enhancing surgical access and minimizing intraoperative bleeding. Relugolix theoretically has a role in endometrial cancer management due to its hormone-suppressing effects, but it is not commonly used due to limited clinical evidence and the preference for more established therapies like progestins, which act directly on endometrial cells to counteract estrogen’s effects rather than merely reducing hormone production [4]. This case is valuable as it illustrates how uterine carcinoma can undergo morphological changes following modification by relugolix, highlighting its potential impact on tumor behavior.

A scoring method based on hysteroscopic appearance was used to detect endometrial malignancy, assigning four points for desquamation, six points for atypical vessels, and one point for white/yellow lesions. Endometrial malignancy was defined with a score of 5 or higher, yielding a sensitivity and specificity of 100% and 92%, respectively [5]. In this case, relugolix caused the polyp with atypical vasculature observed during the previous hysteroscopy to disappear; thus, during surgery, the remaining white, scar-like lesion was interpreted as a shrunken polyp, leading to a failure to recognize the malignancy. 

**Figure 4:**
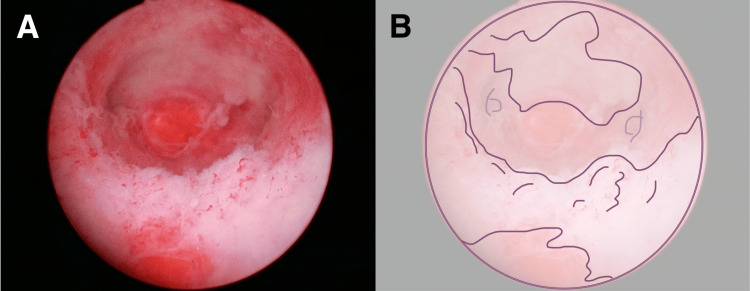
Distinctive findings suggest a malignancy A: The appearance of the grade 2 endometrial cancer after use of the GnRH antagonist under hysteroscopy. The lesion appeared as a flattened, map-like area lining the lower to the circumferential uterine cavity, composed of white, non-translucent tissue with villous-like thickness—an atypical appearance for endometrial polyps. B: Schermer depicts the spread of cancer. GnRH: Gonadotropin-releasing hormone

Because there is no current consensus on whether hysteroscopic perfusate contributes to intraperitoneal tumor seeding in uterine cancer, physicians may have limited familiarity with hysteroscopic findings in such cases [6]. However, retrospectively, we identified some distinctive findings after examining the pathology results and operative video in this case. The lesion appeared as a flattened, map-like area lining the lower to the circumferential uterine cavity, composed of white, non-translucent tissue with villous-like thickness, which is atypical for endometrial polyps (Figure 4). We reflected that the creeping growth pattern that lined the uterus should have led to suspicion of a malignant tumor (Video 1).

**Video 1:** Hysteroscopic findings The lesion appeared as a flattened, map-like area lining the lower to circumferential uterine cavity. It comprises white, non-translucent tissue with villous-like thickness, which is atypical for endometrial polyps. When retrospectively reviewed, the differential diagnosis of malignancy should be raised.

The theoretical therapeutic role against endometrial cancer due to hormone suppression is possibly responsible for the change of the endometrial polyps, albeit with short-term (19 days) relugolix administration in this case. 

Cancers of the LUS are relatively rare, as most endometrial cancers develop in the endometrium of the uterine body. However, when LUS cancer does occur, it is often associated with hereditary cancer syndromes such as Lynch syndrome [7]. Considering that Lynch syndrome is more prevalent in younger patients [8], often overlapping with reproductive age, there is a clinical need to understand GnRH antagonist-induced changes in LUS cancer morphology.

## Conclusions

We were able to observe hysteroscopic findings of grade 2 endometrioid cancer, which was modified by the GnRH antagonist for less than a month. Looking back on the surgery video, the tissue was atypical, thick, white, and expansive, the observation unlike the usual malignant findings or polyp atrophy. Additionally, LUS cancers, particularly those associated with Lynch syndrome, are more prevalent among younger individuals, coinciding with the reproductive age when GnRH antagonists are often administered. Therefore, understanding the morphologic changes induced by GnRH antagonists in LUS cancer is required, particularly in surgeons who operate hysteroscopy.
